# The VIIRS Cirrus Reflectance Algorithm

**DOI:** 10.3390/s23042234

**Published:** 2023-02-16

**Authors:** Bo-Cai Gao, Rong-Rong Li

**Affiliations:** Naval Research Laboratory, Remote Sensing Division, Code 7230, Washington, DC 20375, USA

**Keywords:** remote sensing, sensors, atmosphere, cirrus clouds

## Abstract

The VIIRS instrument (Visible Infrared Imaging Radiometer Suite) on board the SNPP (Suomi National Polar-orbiting Partnership) satellite contains 11 narrow channels (M1–M11) in the 0.4–2.5 μm solar spectral region. The M9 channel is specifically designed for detecting thin cirrus clouds. It is centered at 1.378 μm with a width of 15 nm, which is located within a strong atmospheric water vapor band absorption region. In comparison with the corresponding MODIS Channel 26, the VIIRS M9 channel is narrower and more sensitive for cirrus detections. Because the radiances of the M9 channel over cirrus pixels are subjected to absorption by atmospheric water vapor molecules above and within the cirrus clouds, the water vapor absorption effect needs to be properly taken into consideration when using the M9 channel for quantitative removal of cirrus effects in other VIIRS channels in the 0.4–2.5 μm spectral range. In this article, we describe in detail an empirical technique for the retrieval of cirrus reflectances in the visible and near-IR (VNIR, 0.4–1.0 μm), where ice particles within cirrus clouds have negligible absorption effects, and in shortwave IR (SWIR, 1.0–2.5 μm) where ice particles’ absorption effects are observed. The descriptions include all elements leading to the development of the operational VIIRS cirrus reflectance algorithm, the journal literature backing up the approach, theoretical descriptions of the algorithm’s physics and mathematical background, and sample retrieval results from the VIIRS data. The SNPP VIIRS cirrus reflectance data products from 1 March 2012 to the present are available from a NASA data center.

## 1. Introduction

In Earth-looking satellite visible and thermal IR images, thin cirrus clouds are often not easily seen because of their partially transparent nature. Based on our analysis of hyperspectral imaging data measured by the AVIRIS (Airborne Visible/Infrared Imaging SpectroRadiometer [1,2]) instrument in early 1990s, we observed that narrow channels (~10 nm) located within the 1.38 and 1.88 μm strong atmospheric water vapor absorption bands were sensitive in detecting thin cirrus clouds against a nearly black background [3]. This observation led to the selection and implementation of the MODIS (Moderate Resolution Imaging SpectroRadiometer) [4,5] cirrus detecting channel centered at 1.375 μm with a width of 30 nm [6]. Examples of success in detecting and removing thin cirrus clouds in visible band images using the MODIS 1.375-μm band image were shown during the 19 April 2000 NASA Press Release [7]. As a result, the concept in using the strong water vapor absorption channel for remote sensing of cirrus clouds from space was well accepted by the atmospheric sciences community worldwide. Since then, similar channels for cirrus detections have been implemented from a number of satellite instruments, including the Visible Infrared Imaging Radiometer Suite (VIIRS) [8] on board the Suomi National Polar-orbiting Partnership (SNPP) satellite, MultiSpectral Instrument (MSI) on board the European Sentinel-2 spacecraft [9], and Operational Land Imager (OLI) on board the Landsat 8 satellite [10].

Previously, we described techniques for detecting and correcting thin cirrus effects from hyperspectral and multi-spectral imaging data [11,12,13]. Other researchers also reported methods for cirrus masking and corrections [14,15,16,17]. In this article, we describe the algorithm that has been used by NASA for operational generation of a cirrus reflectance data product from SNPP VIIRS data. The descriptions include all elements leading to the development of the operational VIIRS cirrus reflectance algorithm, the journal literature backing up the approach, theoretical descriptions of the algorithm’s physics and mathematical background, and sample retrieval results from the VIIRS data.

## 2. Background and Methods

### 2.1. Historical Perspective on the 1.38 μm Cirrus Detecting Channel

Historically, the discovery of the 1.38-μm channel for remote sensing of cirrus clouds did not come from the traditional atmospheric sciences research community. As early as mid-1960s, researchers already made spectral measurements in the 1.0–3.0 μm range above cirrus clouds from aircraft platforms [18]. These researchers and many researchers in the atmospheric sciences community later were unable to realize the utility of 1.38-μm channels for cirrus detections. The breakthrough came in early 1990 [6] with the analysis of 3-dimensional hyperspectral imaging data (1-D spectral and 2-D spatial) acquired over cirrus clouds by AVIRIS from an ER-2 aircraft at an altitude of 20 km. The first set of AVIRIS cirrus data was acquired over the Rocky Mountains in March of 1990. Around December of 1990, we were able to view the 3-dimensional AVIRIS data on a Dec 3100 workstation. At the time, we were able to display different band images sequentially on the computer screen. We observed that, cirrus clouds showed up very nicely in images of AVIRIS’s narrow channels (10 nm wide) located within the strong 1.38 μm water vapor band absorption region. Land surface features, such as roads, disappeared in these images. We also observed that, in images of atmospheric ‘window’ channels centered near 1.24 and 1.5 μm, cirrus cloud features were hardly seen while land surface features were very clearly seen. After several days of thinking, we figured out the mechanism for the observation of cirrus and the disappearance of surface features in the 1.38-μm channel images. Because cirrus clouds were high in the atmosphere (~10 km above the sea level), the 1.38 μm solar radiation on the downward sun-surface path was scattered by cirrus clouds and the scattered radiance in the upward path was then detected by the AVIRIS sensor. The 1.38 μm solar radiation transmitted through cirrus clouds in the downward path was absorbed by water vapor beneath the cirrus clouds. As a result, the 1.38-μm channel detected thin cirrus over a nearly ‘black’ background.

**Figure 1:**
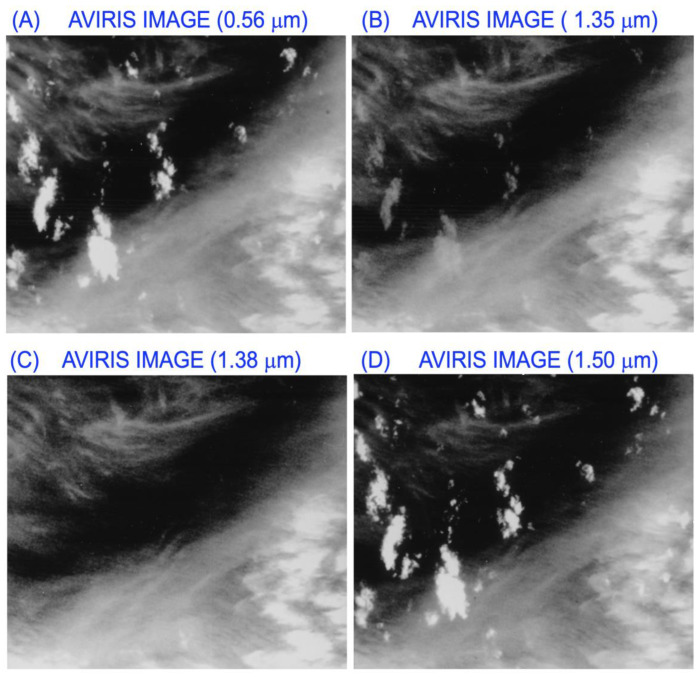
Examples of AVIRIS images for 10 nm wide channels centered at 0.56 (**A**), 1.35 (**B**), 1.38 (**C**), and 1.50 μm (**D**). The images were acquired on 5 December 1991 over the Gulf of Mexico. The image covers an area of approximately 12 by 10 km^2^.

Based on our observations from AVIRIS data and with strong encouragement from NASA scientists and managers, we proposed to fly AVIRIS during a NASA-sponsored major cloud experiment conducted over the Gulf of Mexico, Kansas, and Oklahoma areas in November and December of 1991. AVIRIS was placed onto an ER-2 aircraft only for the last three days of the experiment, and collected excellent data sets on 5 and 7 December. Figure 1 shows examples of the AVIRIS images acquired over the Gulf of Mexico on 5 December 1991. In the 0.56- and 1.50-μm atmospheric ‘window’ channel images, both the upper level cirrus clouds and the lower level brighter and isolated cumulus clouds were seen. In the 1.38-μm channel image, only the upper level cirrus clouds were seen. The lower level cumulus clouds disappeared completely because of strong water vapor absorption of solar radiation transmitted through the cirrus clouds in the downward sun-surface path. In the 1.35-μm channel image, weak cumulus cloud features were seen. This is because the atmospheric water vapor absorption effect was not sufficiently strong at this wavelength to result in total absorption of solar radiation beneath the cirrus clouds.

During the NASA MODIS Science Team meeting held near Santa Barbara, California in December 1992, we presented the AVIRIS cirrus images and proposed to implement a cirrus detecting channel on MODIS. The MODIS Science Team endorsed the idea of putting a narrow channel centered near 1.38 μm [6] on MODIS. With strong support from the MODIS scientists and the management team at NASA’s Goddard Space Flight Center, it was possible to implement this cirrus detecting channel (Band 26) onto MODIS during the very late stage of the MODIS instrument design. However, it resulted in the loss of an originally designed MODIS CO_2_-slicing channel centered at 4.565 μm [19].

### 2.2. The VIIRS Instrument and the M9 Cirrus Detecting Channel

The VIIRS instrument is similar to the MODIS instruments currently on board the NASA Terra and Aqua Spacecrafts. The VIIRS channel names, positions, and widths are listed in Table 1. Many VIIRS channels (designated as M1 to M16 in Table 1) have heritages to MODIS but with minor differences in center positions and widths. Important differences between VIIRS and MODIS do exist. For example, MODIS channels located in atmospheric gaseous absorption regions near 4.5 μm, 6.7 μm, and above 13 μm are all absent in VIIRS. As a result, VIIRS has, in general, less capability for remote sensing of atmospheric temperatures and clouds in comparison with MODIS.

**Figure 2:**
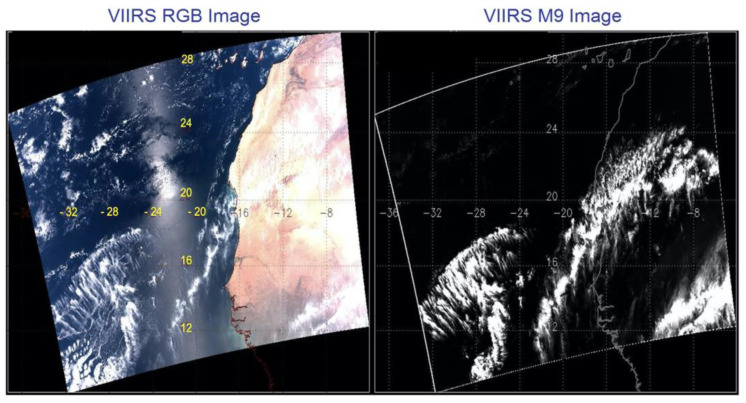
An example of VIIRS images over desert and water areas affected by sunglint.

Fortunately, VIIRS has implemented the M9 channel centered at 1.378 μm with a width of 15 nm for remote sensing of cirrus clouds from space. Soon after the launch of the Suomi VIIRS instrument into space, we evaluated the quality of the M9 channel data. The left plot in Figure 2 shows a VIIRS RGB image acquired over bright desert and dark ocean water areas. Sunglint patterns in the left portion are clearly seen. The right plot in Figure 2 shows the corresponding VIIRS M9 channel image. Thin cirrus clouds over the bright desert and water surfaces are nicely detected, while the sunglint features are not seen at all. The Figure 2 images demonstrated the great capability of the VIIRS M9 channel for cirrus detections over water, land, as well as areas affected by sunglint.

### 2.3. Absorption and Scattering Properties of Cirrus Clouds

**Figure 3:**
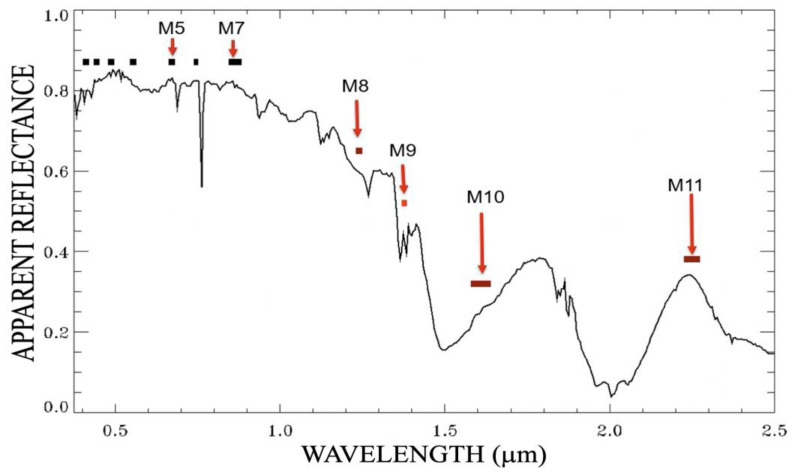
A thick cirrus apparent reflectance spectrum. The VIIRS M1–M7 channels are marked in short black bars. The M8–M11 channels are marked separately in short red bars [20], because the four SWIR bands have ice absorption effects.

Ice particles within cirrus clouds have a variety of sizes and shapes. The effective particle sizes are usually larger than 5 µm. We illustrate the scattering and absorption properties of cirrus clouds through recent hyperspectral imaging data acquired with the next generation of AVIRIS (nicknamed as AVIRIS-NG) from an ER-2 aircraft at an altitude of 20 km. Figure 3 [20] shows a sample ‘apparent reflectance’ spectrum measured over an area covered by thick cirrus clouds with AVIRIS-NG from ER-2. This figure was previously included in a NASA report entitled ‘VIIRS Suomi-NPP Level-2 Cirrus Reflectance Product (CLDCR_L2_VIIRS_SNPP) User Guide’ [20]. Here, apparent reflectance represents the ratio of measured radiance over the incoming solar irradiance. The VIIRS M1–M11 channel positions and widths are marked in short and thick horizontal bars. In this cirrus spectrum, the atmospheric water vapor absorption bands centered near 0.94, 1.14, 1.38, and 1.88 μm were seen. The narrower atmospheric oxygen bands centered near 0.69, 0.76, and 1.26 μm were also seen. In addition, a broad atmospheric ozone absorption band (Chapius band) centered near 0.60 μm was seen. For the cirrus spectrum, the reflectances of ice particles in the 0.4–1.0 µm spectral region were nearly constant with wavelength, because ice particles are much larger than the wavelength and are non-absorbing in this spectral range. Past 1.0 µm, one finds several ice absorbing bands, for example those centered near 1.5 and 2.0 µm. Both the M10 (1.61 μm) and M11 (2.25 μm) channels were affected by ice absorption effects. Because M11 is centered near a local reflectance maximum, the overall ice absorption effect for the M11 channel can be smaller than that of the M10 channel. Weak ice absorptions occurred near 1.24 µm (M8) and 1.375 µm (M9); the imaginary parts of the ice refractive index were about the same at both wavelengths. The measured reflectances at 1.375 µm were smaller than those in the 0.4–1.0 µm region mainly because of absorption by water vapor above and within the cirrus clouds. These high-altitude water vapor absorption effects need to be accounted for in order to use the VIIRS M9 channel for quantitative retrieval of cirrus reflectances and for subsequent removal of cirrus effects in the M1–M8 channels. The use of the M9 channel for the removal of cirrus effects in the M10 and M11 channels needs to take into considerations the M9 water vapor absorption effects and the M10 and M11 ice absorption and scattering effects.

### 2.4. Method for Retrieving Cirrus Reflectances from VIIRS Data

Cirrus clouds contain mainly ice particles and are located in the upper troposphere and lower stratosphere. As far as the cirrus reflection and scattering effect is concerned, we can assume that a homogeneous thin cirrus layer is located above a “virtual surface”, which includes the effects of scattering by molecules, aerosols, and low clouds as well as land or ocean surface reflection and sub-surface scattering. Omitting, for convenience, the wavelength (λ) and cosine solar zenith angle (*μ*_0_) dependencies, we can denote the “apparent reflectance” at the satellite as:

(1) $$\rho *=\frac{\pi \;L}{\mu_{0}E_{0}},$$

where *L* is the radiance measured by the satellite, *μ*_0_ is the cosine of solar zenith angle, and *E*_0_ is the extra-terrestrial solar flux. In general, ρ* consists of the cirrus reflection component (*ρ*_C_) and the virtual surface reflection component (*ρ*_S_).

As described above, the solar radiance within the VIIRS M9 cirrus band is partially absorbed by water vapor molecules located above and within cirrus clouds (see Figure 3). It is practically difficult to quantitatively derive both the upper level water vapor transmittance factor and the cirrus reflectance on a pixel-by-pixel basis from the 1.375 µm (M9) cirrus image alone, i.e., it is not possible to retrieve two unknowns from one measurement. In view of this situation, we have decided to obtain the correlation between a given band image and the 1.375-µm cirrus image [11,12]. We then use the correlation and the 1.375-µm cirrus image to obtain the cirrus reflectance image of a given band. In this way, the information contained in the spatial domain of a scene is used for the derivation of a mean value of upper level water vapor transmittances of the scene.

**Figure 4:**
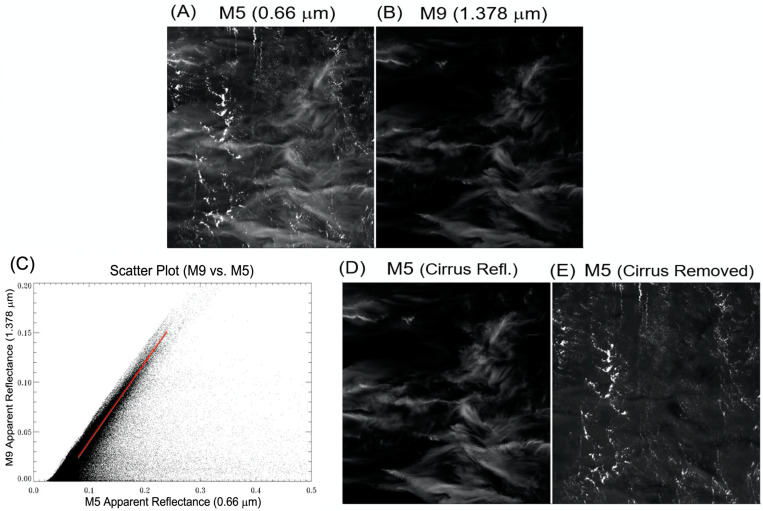
A portion of a VIIRS M5 band apparent reflectance image (**A**), the M9 band apparent reflectance image (**B**), the scatter plot of M9 versus M5 band images (**C**), the derived M5 band cirrus reflectance image (**D**), and the cirrus-removed M5 band apparent reflectance image (**E**) [20].

Below we use a VIIRS scene to illustrate the cirrus reflectance derivation and the subsequent cirrus correction processes. Figure 4A is a portion (about 530 by 530 pixels) of a VIIRS M5 (0.672 μm) apparent reflectance image. Thin cirrus clouds and lower level brighter water clouds are seen. Figure 4B is the corresponding M9 apparent reflectance image. Only the upper level cirrus clouds are seen. Figure 4C is the scatter plot of the apparent reflectance images of M9 versus M5 bands. Pixels with the least surface and lower level water cloud reflection contributions are located near the left edge portion of the scatter plot along a straight line. These pixels were used for the estimation of the slope (shown as red line in the plot). During the slope estimation process, bad pixels with negative reflectance values or fill values were first eliminated. Very bright pixels with M5 apparent reflectance values greater than 1.0 were also eliminated. The data points along the vertical axis (M9 apparent reflectance) were divided into 20 layers. For each of the layers, the data points were sorted according to the apparent reflectance values of the M5 band from low to high. In order to eliminate possible additional noisy and bad pixels, 5% of pixels in the lower end of M5 band apparent reflectance values were rejected. The next 5% of the pixels were used to calculate mean values of the apparent reflectances for the M5 and M9 bands for a given layer. For the 20 layers, we had a total of 20 pairs of mean M5 and M9 apparent reflectances. The 20 data pairs were then used for the estimation of a mean slope value, as illustrated in the thick red line in Figure 4C. Figure 4D is the retrieved M5 band cirrus reflectance image, which is brighter than the Figure 4B M9 apparent reflectance image. This is because the estimated slope value was smaller than 1.0, and the division of the M9 image by the slope value increased the M5 band cirrus reflectance value. In order to test if the estimated M5 cirrus reflectances are correct, we show in Figure 4E the cirrus-corrected M5 band apparent reflectance image. By comparing Figure 4E with Figure 4A, it could be seen that cirrus cloud features were properly removed in the Figure 4E image. This demonstrates that the derived M5 band cirrus reflectances were sufficiently accurate for pixel-by-pixel cirrus removals.

A summary of the procedures for deriving cirrus reflectances and for removing cirrus scattering effects of a given band (*B*) using the information contained in the 1.38 µm band (M9, cirrus band) has previously been given by Gao and Li [13]. The steps include:

(a) Converting the measured radiances (*L*) into apparent reflectances (ρ*) using Equation (1);
(b) Generating the scatter plot of ρ*(cirrus) versus ρ*(*B*) (e.g., Figure 4C);
(c) Estimating the slope, *S*_B_, from the scatter plot (also see Figure 4C);
(d) Calculating the cirrus reflectance of the given band, $\rho_{C}(B)$, which is equal to ρ*(cirrus)/*S_B_*;
(e) Subtracting out the cirrus reflectance, $\rho_{C}(B)$, from the measured apparent reflectance, ρ*(*B*), for removing the cirrus scattering effect in band *B*.

The steps described above are applicable for the correction of cirrus scattering effects of any given band in the 0.4–2.5 µm solar spectral range, regardless of whether the band having ice absorption effects or not. For some scenes, there were no cirrus clouds at all. It was not possible to obtain reliable slopes from the scatter plots (e.g., Figure 4C). Under such circumstances, we assigned default slope values based on vertical distributions of atmospheric water vapor in climatological model atmospheres, and the solar and viewing angles.

### 2.5. Algorithm Implementation

**Figure 5:**
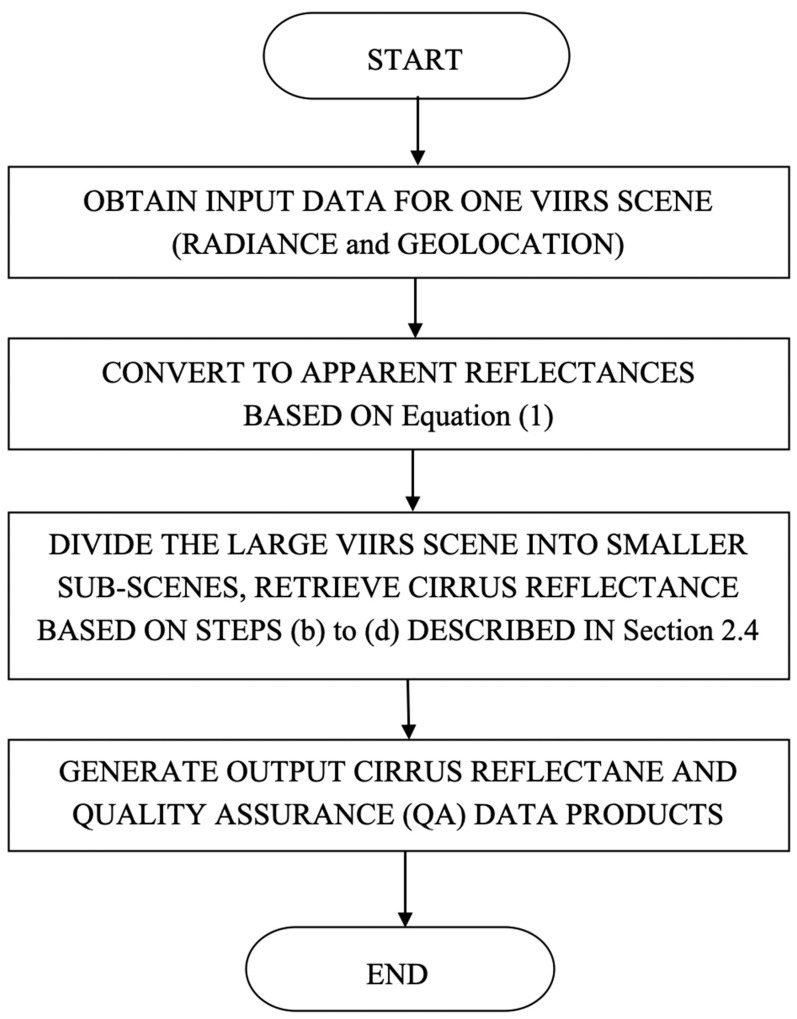
A flow chart describing the implementation of the VIIRS cirrus reflectance algorithm.

Figure 5 is a flow chart illustrating the procedures for implementing the VIIRS cirrus reflectance algorithm. Typically, the spatial area covered by a standard VIIRS 6-min granule is more than 3000 × 2000 km^2^. Over such a large scene, we cannot assume that the upper level water vapor distributions are spatially homogeneous. To overcome this problem, we often divide a whole VIIRS scene into 6 × 6 smaller sub-scenes. Prior to the selection of this scene division scheme, we also tried the divisions into 4 × 4, 5 × 5, 7 × 7, and 8 × 8. We found that the 6 × 6 division avoided the spatial inhomogeneous problem associated with the upper level water vapor distributions and retained the computational efficiency for massive operational VIIRS cirrus reflectance retrievals.

**Figure 6:**
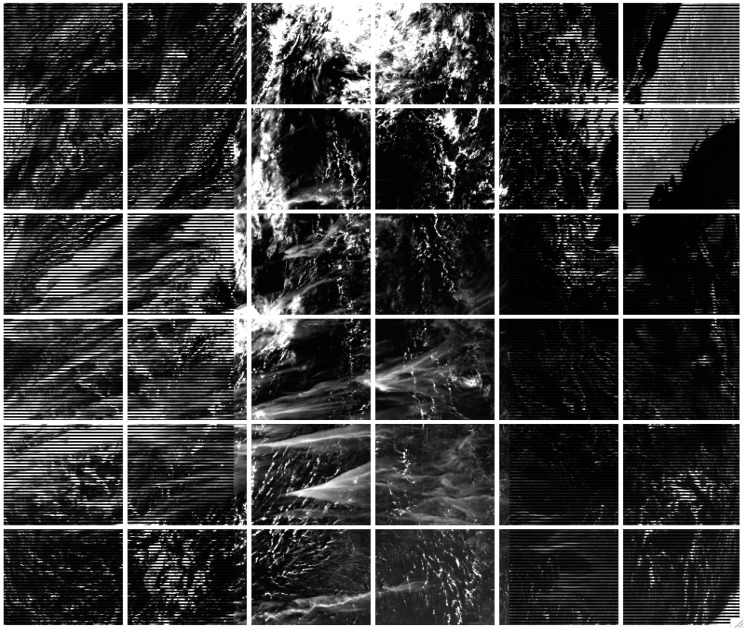
A VIIRS M5 (0.672 μm) image acquired on 10 July 2017 at UTC 0936. The complete scene is divided into 6 × 6 smaller sub-scenes during the cirrus reflectance retrieving process.

Figure 6 shows an example of a VIIRS M5 (0.672 μm) channel image (acquired on 10 July 2017 at UTC 0936), where the complete scene was divided into 36 smaller scenes. For each sub-scene, we used the scatter plot approach, as illustrated in Figure 4C, to derive a mean slope value for a given VIIRS VIS-NIR band, or a SWIR band. Using 2-dimensional linear interpolation and extrapolation techniques [12], we then obtained slope values (*S_B_*) of a given band for the entire scene on a pixel-by-pixel basis from the 36 mean slope values. The pixel-by-pixel-based cirrus reflectances, $\rho_{C}(B)$, were finally obtained through the equation:

(2) $$\rho_{C}(B)=\rho *(M9)/S_{B},$$

where ρ*(M9) is the apparent reflectances of the VIIRS M9 band. To make cirrus correction, we subtract out the cirrus reflectance, $\rho_{C}(B)$, from the measured apparent reflectance, ρ*(*B*). The resulting cirrus-corrected apparent reflectance for band B, ρ*(*B_Corr*), was obtained according to the following equation:

(3) $$\begin{array}{cc} \rho *(B\_\mathrm{Corr}) & =\rho *(B)-\rho_{C}(B)\\ & =\rho *(B)-\rho *(M9)/S_{B} \end{array}$$

### 2.6. Quality Assurance

**Figure 7:**
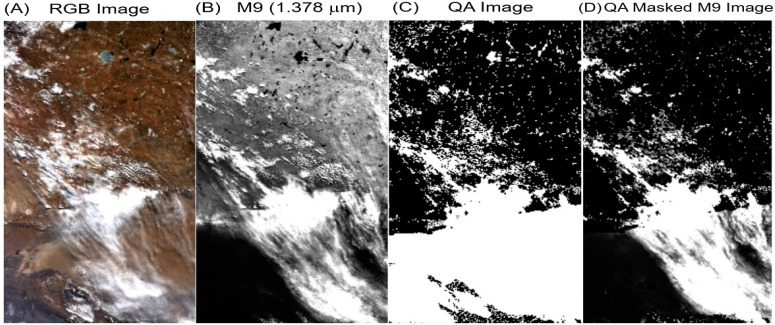
A portion of a VIIRS RGB image over Tibet Plateau (**A**), the M9 band apparent reflectance image (**B**), the QA image (**C**), and the QA masked M9 band image (**D**).

Under typical atmospheric conditions with a column amount of atmospheric water vapor at 0.4 cm or larger, there is sufficient amount of water vapor in the lower level of the atmosphere to cause total absorption of solar radiation near 1.38 μm in the sun–surface–sensor ray path. The VIIRS M9 channel detects the solar radiation scattered by the upper level cirrus clouds without contaminations from the bottom surfaces. However, under very dry atmospheric conditions with a column amount of water vapor at 0.1 to 0.2 cm, the M9 channel also receives solar radiation reflected and scattered by the earth’s surfaces. Figure 7A shows a portion of a VIIRS RGB image acquired over the high elevation Tibet Plateau during a dry season. Clouds and surface features are observed. Figure 7B shows the corresponding M9 channel image. Weak land features over clear surface areas are present. Figure 7C is a QA (Quality Assurance) image we generated after applying a variety of criteria (to be described below) to the VIIRS data set. In this QA image, pixels with poor qualities were assigned a value of zero. Pixels with high qualities were assigned a value of 2. The remaining pixels are assigned a QA value of 1. Figure 7D is the M9 image after application of a mask based on QA values of the pixels in the scene. By comparing Figure 7D with Figure 7B, it can be seen that most land features were successively masked out in Figure 7D.

We have made major efforts in developing the QA routine used in the ‘operational’ VIIRS cirrus reflectance algorithm. In the present implementation, for a pixel within the high elevation Tibet Plateau with latitudes between 27 and +45 degrees, longitudes between 70 and 100 degrees, surface elevation between 1500 and 3000 m, the apparent reflectance of the M9 channel was less than 0.12, and the apparent reflectance of M8 (1.24 μm) was greater than that of M5 (0.672 μm), the pixel is assigned a QA value of zero. The last requirement, in particular, is essential in assigning the small QA value for the high reflecting land pixel. However, if the additional test showed that the apparent reflectance of M8 (1.24 μm) is less than 0.08 (such as a high elevation lake), the QA of the pixel is re-assigned to a value of 2, i.e., the pixel is not a bright land pixel. The QA routines for the high elevation Andes Mountains and Rocky Mountains were similar to that for the Tibet Plateau, except for minor differences in threshold values. It should be pointed out that our selection of threshold values was made after many tests with multiple VIIRS data sets acquired over different geographical regions in different seasons. Initially, we also tried to use VIIRS IR emission bands, such as M15 and M16, and to set threshold values for these bands’ brightness temperatures for the assignment of QA values; however, we found that less consistent results were obtained. Further tests using VIIRS IR emission channels to improve QA parameter assignment are needed.

For polar regions with solar zenith angles greater than 88 degrees, we no longer made cirrus reflectance retrievals. The corresponding pixels’ cirrus reflectance values were set to zero, and the QA values were also set to zero. For pixels with solar zenith angles less than or equal to 88 degree and with a QA value equal to zero (e.g., very dry high elevation mountain pixels such that the M9 band receives small amounts of solar radiation reflected by the bottom surfaces), these pixels’ cirrus reflectances were reset to the M9 band’s apparent reflectances. End users of the VIIRS cirrus reflectance products are recommended to mask out these pixels before making quantitative use of the data products.

### 2.7. Descriptions of Input and Output Data Products

The VIIRS Level 2 cirrus reflectance algorithm requires input data sets from the standard Level 1b calibrated radiance and geolocation data cubes in netCDF4/HDF5 format. Specifically, the input data for a given VIIRS scene include: number of samples (the data points in the scan direction), number of lines (the data points in the flight direction), the apparent reflectance of M5 (0.672 μm), M8 (1.24 μm), M9 (1.378 μm), M10 (1.61 μm), and M11 (2.25 μm) channels; the brightness temperature of M14 (8.55 μm), M15 (10.76 μm), and M16 (12.01 μm) channels; latitude, longitude, and surface elevation; and solar zenith angle, solar azimuth angle, view zenith angle, and view azimuth angle. The output data for a given VIIRS scene include: cirrus reflectance for visible and near-IR (VNIR) channels, cirrus reflectances for shortwave IR (SWIR) channels centered at 1.24, 1.61, and 2.25 μm, and the associated QA (quality assurance) parameter.

### 2.8. Variance and Uncertainty Estimates

By varying different parameters used in our cirrus reflectance algorithm, we typically get consistency in the derived slope values (see for example Figure 4C) to within about 2%. Suppose for a thin cirrus pixel with the M9 apparent reflectance value to be 0.025 and the estimated slope value to be 0.5, the estimated cirrus reflectance uncertainty for this pixel would be about 0.02 times 0.025 and then divided by 0.5, which is equal to 0.001. The use of this cirrus reflectance data product in subsequent cirrus corrections would introduce the same amount of error in the derived surface reflectance data product. We feel that our retrieved VIIRS cirrus reflectance data products are suited for use in cirrus corrections, as demonstrated in Figure 4E.

### 2.9. Programming and Procedural Considerations

The central portion of the VIIRS cirrus reflectance algorithm is written in Fortran90. This portion of the code is almost machine-independent and portable to any computers, as long as the computer has an F90 compiler. The input and output interface to access the VIIRS radiance and geolocation data sets in netCDF4/HDF5 and to output the cirrus reflectance data product also in netCDF4/HDF5 is written in ‘c’. The ‘c’ routines can have minor portability issues with different computers having different operating systems.

## 3. Results

At present, global VIIRS cirrus reflectance data products have been operationally produced at a NASA data center. Below we show a few cases on cirrus retrievals and cirrus corrections from individual VIIRS granules. We also show sample global cirrus reflectance images for a few days in different seasons.

### 3.1. Cirrus Corrections—Red Sea, 1 January 2013

**Figure 8:**
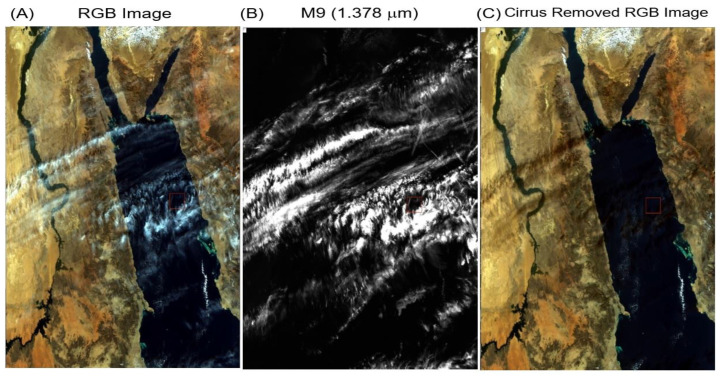
A portion of a VIIRS RGB image over Red Sea and nearby areas (**A**), the M9 band apparent reflectance image (**B**), and the cirrus-removed RGB image (**C**) [20].

Figure 8A is a portion of a VIIRS RGB image acquired on 1 January 2013 over the Red Sea area. The white cirrus features over land and water surfaces are seen. Figure 8B is the corresponding M9 (1.378 μm) cirrus image. Here, both the land and water surface features were seen because of strong absorption near 1.378 μm by water vapor beneath the cirrus clouds. Figure 8C is the cirrus-removed RGB image. The white cirrus features seen in Figure 8A disappeared completely in the Figure 8C image, which indicates the proper removal of thin cirrus scattering effects using the derived cirrus reflectances. In addition, the small greenish-colored water features in Red Sea were observed more clearly than those in the Figure 8A original RGB image. The small, bright, cumulus clouds in the upper left portion and lower central portion of the scenes remained in the Figure 8C image. There were dark features in the Figure 8C image over desert areas. These features are related to the cirrus shadows in the Figure 8A RGB image, and they were not results of over-correction of the cirrus effects. These dark features were spatially displaced in comparison with the corresponding cirrus features in Figure 8B.

To show more quantitatively the importance of cirrus removals, we calculated the mean apparent reflectance for the VIIRS M7 (0.865 μm) band, which is dark over water surfaces, for the Figure 8 scene before the cirrus removal. The mean value was 0.254. After the cirrus removal, the mean value became 0.229—a decline in reflectance for the scene by 9.8%. For the darker water surfaces, the mean apparent reflectance before cirrus removal was 0.050. The mean value after the cirrus removal was 0.021—a decrease in reflectance over the water surfaces by 58%. Therefore, it is more important to make cirrus corrections over dark water surfaces than over bright land surfaces.

### 3.2. Cirrus Corrections—Black Sea, 30 May 2022

**Figure 9:**
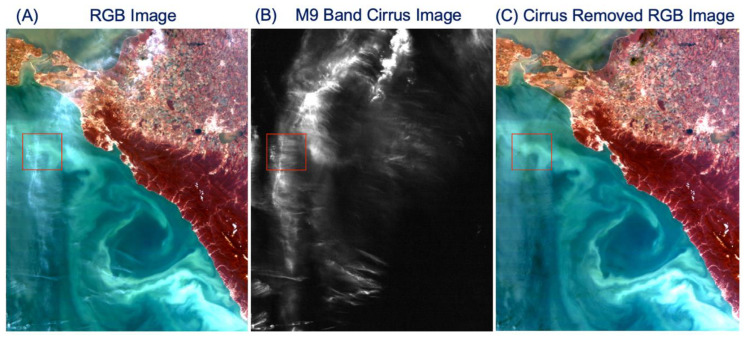
A portion of a VIIRS RGB image over eastern part of Black Sea and nearby areas (**A**), the M9 band apparent reflectance image (**B**), and the cirrus-removed RGB image (**C**).

Figure 9A shows a portion of a VIIRS RGB image measured on 30 May 2022 in the eastern part of the Black Sea and nearby land areas. Complex and colored spatial patterns over water surfaces are seen. Figure 9B is the M9 cirrus image. Figure 9C is the cirrus-removed RGB image using the retrieved cirrus reflectance data product. By comparing the three images, it can be seen that the spatial distribution patterns over water surfaces were blurred by cirrus clouds in the original RGB image. After the cirrus removal, the spatial patterns over waters were significantly improved, for example over the small area within the red box. In addition, cirrus clouds over land areas were also removed. For this scene and for all water pixels, the mean reflectance value after the cirrus removal was 18% smaller than that before the removal. This shows again the importance in making cirrus corrections over dark surfaces.

### 3.3. Cirrus Reflectances—Southern Atlantic Ocean, 1 September 2016

**Figure 10:**
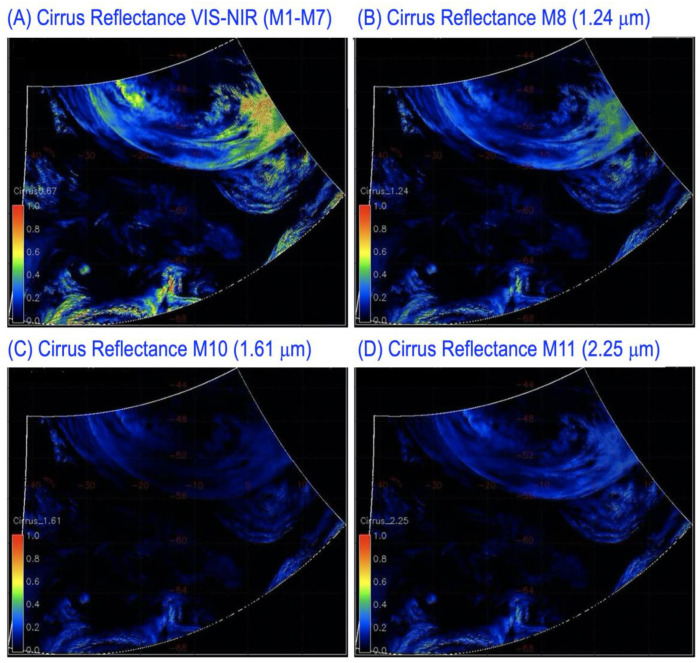
Sample retrievals of cirrus reflectances for VIIRS VNIR channels (M1–M7) (**A**), SWIR channels M8 (1.24 μm) (**B**), M10 (1.61 μm) (**C**), and M11 (2.25 μm) (**D**) for one 6 min VIIRS granule acquired over southern Atlantic Ocean on 1 September 2016.

Figure 10 shows examples of retrieved cirrus reflectance images for one 6 min VIIRS granule acquired at UTC 15:18:00 on 1 September 2016 over southern Atlantic Ocean. Figure 10A–D are the images for VIIRS VIS-NIR channels (M1–M7), SWIR channels M8 (1.24 μm), M10 (1.61 μm), and M11 (2.25 μm), respectively. These images are false color-coded with the same color scale. Deep blue corresponds to a cirrus reflectance of 0.0. Bright red corresponds to a cirrus reflectance of 1.0. One color bar representing the false color scale is placed inside each of the images. These images were mapped using the so called Mercator projection. The mapped images covered the latitude range of 42°–68° S and the longitude range of 40° W–20° E. Excellent cirrus spatial distribution patterns were seen in these images. By comparing Figure 10C with Figure 10D, it can be seen that the M10 (1.61 μm) cirrus reflectance values were smaller than those of the M11 (2.25 μm). This is because the ice particles within cirrus clouds can have stronger absorption effects over the M10 bandpass than over the M11 bandpass (see also the cirrus spectrum in Figure 3).

### 3.4. Global Cirrus Reflectance Images—1 January, 1 April, 1 July, and 1 October 2021

**Figure 11:**
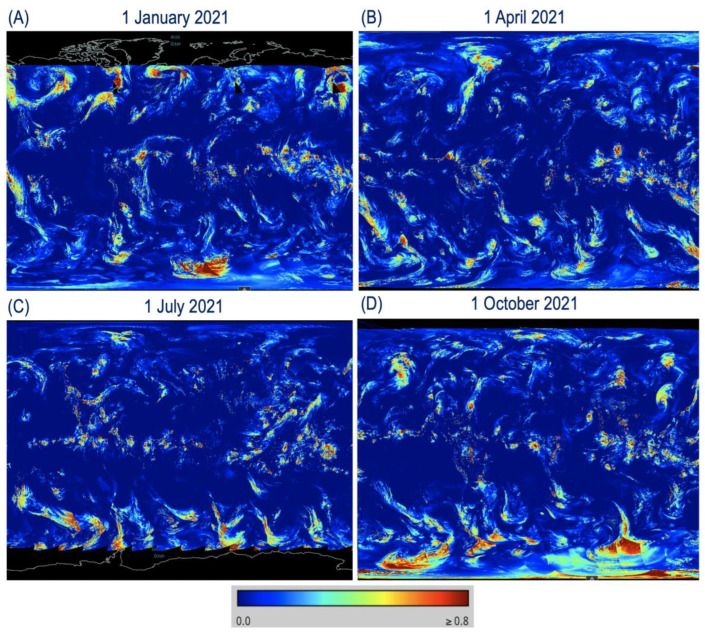
Global VIS-NIR cirrus reflectance images for 1 January (**A**), 1 April (**B**), 1 July (**C**), and 1 October (**D**) of 2021.

Global VIIRS cirrus reflectance imaging products for VNIR bands and for SWIR M11 band (2.25 μm) from 1 March 2012 to the present can be viewed from the website https://worldview.earthdata.nasa.gov (accessed on 3 January 2023). Below, we give detailed steps for facilitating the easy access of VIIRS cirrus reflectance images from this NASA website, particularly for new data users. After connecting to this website and clicking the red-colored button ‘+Add Layers’ in the lower left portion of the displaying window, a new window with buttons marked by ‘Hazards and Disasters’ and ‘Science Disciplines’ near the top of the window will pop out. The ‘Cirrus Reflectance’ imaging product can be selected from the ‘Other’ sub-section within the ‘Hazards and Disasters’ section. It can also be selected from the ‘Atmosphere’ sub-section within the ‘Science Disciplines’ section. After the selection of ‘Cirrus Reflectance’, the global cirrus reflectance images for any given day can be displayed on a computer screen. Figure 11 shows sample global VIS-NIR cirrus reflectance images for 1 January (Figure 11A), 1 April (Figure 11B), 1 July (Figure 11C), and 1 October (Figure 11D) of 2021. The spatial distribution patterns of cirrus clouds for the different days and in different seasons are very different. For example, due to the lack of sunshine in winter time, the cirrus reflectance product cannot be produced at high latitude Arctic (see Figure 11A) and Antarctic regions (see Figure 11C).

## 4. Discussions

At present, more detailed information about the VIIRS cirrus reflectance data products can be found from the web link [21] associated with the NASA LAADS DAAC (Lavel-1 and Atmosphere Archive and Distribution System Distributed Active Archive Center). From there, additional web links to the product user guide, to algorithm theoretical basis document (ATBD), for searching and downloading product files, to data archives, and to the dataset’s DOI (Digital Object Identifier) can be found. The information may be helpful to potential scientists who may use the VIIRS cirrus reflectance data products for research and applications.

## 5. Summary

We have described an algorithm for retrieving cirrus reflectances in the visible and near-IR (VNIR, 0.4–1.0 μm) and shortwave IR (SWIR, 1.0–2.5 μm) spectral regions from VIIRS data. The descriptions included all elements leading to the development of the operational algorithm, the journal literature backing up the approach, theoretical descriptions on the algorithm’s physics and mathematical background, and sample retrieval results from the VIIRS data. The cirrus reflectance products can be used for removing cirrus scattering effects for all VIIRS bands located in the 0.41–2.25 micron solar spectral range. The SNPP VIIRS cirrus reflectance data products from 1 March 2012 to the present are publicly available from the NASA LAADS DAAC.

## Figures and Tables

**Table 1:** VIIRS channel names, positions, and full widths at half maximum (FWHMs).

VIIRS Channel	λ (µm)	FWHM (µm)
M1	0.412	0.020
M2	0.445	0.018
M3	0.488	0.020
M4	0.555	0.020
M5	0.672	0.020
M6	0.746	0.015
M7	0.865	0.039
M8	1.24	0.020
M9	1.378	0.015
M10	1.610	0.060
M11	2.250	0.050
M12	3.700	0.180
M13	4.050	0.155
M14	8.550	0.300
M15	10.7625	1.000
M16	12.0125	0.950

## Data Availability

All the data used in this study are available from NASA data centers.
